# Magnetic Resonance Thermometry at 7T for Real-Time Monitoring and Correction of Ultrasound Induced Mild Hyperthermia

**DOI:** 10.1371/journal.pone.0035509

**Published:** 2012-04-20

**Authors:** Brett Z. Fite, Yu Liu, Dustin E. Kruse, Charles F. Caskey, Jeffrey H. Walton, Chun-Yen Lai, Lisa M. Mahakian, Benoit Larrat, Erik Dumont, Katherine W. Ferrara

**Affiliations:** 1 Department of Biomedical Engineering, University of California, Davis, Davis, California, United States of America; 2 Biophysics Graduate Group, University of California, Davis, Davis, California, United States of America; 3 NMR Facility and Biomedical Engineering Graduate Group, University of California, Davis, Davis, California, United States of America; 4 Institut Langevin, ESPCI Paristech, CNRS UMR7589, INSERM, Paris, France; 5 Image Guided Therapy, Pessac, France; City of Hope National Medical Center and Beckman Research Institute, United States of America

## Abstract

While Magnetic Resonance Thermometry (MRT) has been extensively utilized for non-invasive temperature measurement, there is limited data on the use of high field (≥7T) scanners for this purpose. MR-guided Focused Ultrasound (MRgFUS) is a promising non-invasive method for localized hyperthermia and drug delivery. MRT based on the temperature sensitivity of the proton resonance frequency (PRF) has been implemented in both a tissue phantom and *in vivo* in a mouse Met-1 tumor model, using partial parallel imaging (PPI) to speed acquisition. An MRgFUS system capable of delivering a controlled 3D acoustic dose during real time MRT with proportional, integral, and derivative (PID) feedback control was developed and validated. Real-time MRT was validated in a tofu phantom with fluoroptic temperature measurements, and acoustic heating simulations were in good agreement with MR temperature maps. In an *in vivo* Met-1 mouse tumor, the real-time PID feedback control is capable of maintaining the desired temperature with high accuracy. We found that real time MR control of hyperthermia is feasible at high field, and *k*-space based PPI techniques may be implemented for increasing temporal resolution while maintaining temperature accuracy on the order of 1°C.

## Introduction

Magnetic Resonance Thermometry (MRT) has been shown to be a reliable technique for non-invasive, real-time temperature monitoring [1], [2], [3], with temperature measurement implemented in multiple forms [4], [5], [6], [7], [8], [9]. Currently, the most widely used implementations, especially at field strengths above 1T [10], exploit the temperature sensitivity of the proton resonance frequency (PRF) of water molecules. The PRF has a thermal coefficient that is nearly independent of tissue type (with the exception of adipose tissue) while exhibiting linearity over temperatures of physiological interest [8] and therefore can assess temperature change even after tissue coagulation [11]. Coupled with the excellent soft tissue anatomical information provided by MRI, the ability to make accurate measurements of temperature change makes MRI an excellent tool for guiding thermal therapies.

High temporal resolution is often desirable in guiding thermal therapies to ensure that target temperatures are accurately controlled within the treated region while avoiding thermal damage to surrounding healthy tissue. Partial parallel imaging (PPI) techniques using phased arrays have been used for decreasing MR acquisition time during MRT [12], [13], [14]. Both generalized autocalibrated partially parallel acquisition (GRAPPA), and *k*-space inherited parallel acquisition (KIPA) [15] have been utilized for MRT. With large time reduction factors, KIPA exhibits a clear advantage in image quality [14]; however, the ease of use of “push-button” GRAPPA implementations make it an attractive technique when smaller reduction factors (e.g. R = 2) are employed.

Mild-hyperthermia of neoplastic tissues, where temperatures are elevated to between 39°C–42°C, results in increased blood flow [16], increased vascular permeability [17], decreased pH [18], and increased oxygenation [19]. Some novel therapies based on mild hyperthermia, including heat-activated gene therapy [20] and targeted drug delivery via temperature sensitive liposomes (TSL) [21], [22], rely on manipulation of temperature in a region of interest (ROI) to enhance their localization. Precise three dimensional control of therapeutic focused ultrasound (FUS) intensity can be exploited to enhance cancer drug delivery when coupled with circulating TSL with chemotherapeutic cargoes. The ability of MR-guided FUS (MRgFUS) to provide both real-time 3D anatomical information, and temperature data makes the combination especially attractive for precise, localized release of liposomal drugs without inducing thermotolerance typical of higher temperature thermal therapies [23].

The advantages of MRgFUS as a form of image guided therapy, especially for small animal applications, are augmented at higher magnetic field strengths. Namely, the quasi linear increase in both effective signal-to-noise ratio (SNR) and the PRF temperature sensitivity with increasing field strength promise improvements in image quality and temperature precision for MRT at high field strengths (≥7T). High field scanners are particularly advantageous for small animal imaging where the increased SNR as compared with lower field clinical scanners (e.g. 1.5, or 3T) can be utilized to gain needed spatial resolution. Clinical implementations of therapeutic FUS, typically around 1 MHz [24], [25], may have inadequate depth-of-field (∼1 cm) for use in small animals where the focal spot length can be prohibitively large. Thus, benefit is derived from higher frequency FUS where the depth-of-field is significantly reduced and focal spot length is on the order of 1–2 mm (for 3 MHz) [26]. The combination of a 7T scanner and 3 MHz FUS promises to provide numerous advantages for small animal MRgFUS. Moreover, GRAPPA based PPI algorithms provided in many modern MR imagers offer an ease of implementation that is especially attractive if suitable temperature accuracy may be obtained. Thus, we examined the use of GRAPPA for MRT and its applicability on a 7T system for MRT, coupled with a 3 MHz FUS transducer, for use with small animals. We report the development of a combined FUS-MRI system at high field for the delivery of precise thermal doses along arbitrary trajectories facilitated by integrated real-time feedback control. High thermal accuracy, spatiotemporal resolution, and homogeneity of thermal dose distribution were achieved with implementation of GRAPPA.

## Materials and Methods

### Ethics Statement

All animal experiments were performed under a protocol approved by the Institutional Animal Care and Use Committee (IACUC) of the University of California, Davis (protocol # 15717). Anesthesia was administered for all imaging and surgical procedures. All mice were housed in accordance with approved IACUC protocols.

### MRI setup

Imaging studies were performed on a Bruker BioSpec 7T small animal system (Biospec 70/30 USR, Bruker BioSpin, Ettlingen, Germany). Radio frequency (RF) coils used for this study included a circularly polarized RF resonator, internal diameter 154 mm, for ^1^H imaging operating in transmit and receive mode, a cross-coil configuration with a 4-channel rat brain phased array for RF receive, or a cross-coil configuration with a 20 mm diameter circular surface coil for RF receive. The 154 mm volume resonator was used for RF transmit in all cases and data were acquired under Paravision 5.1 in all experiments.

### FUS setup

**Figure 1:**
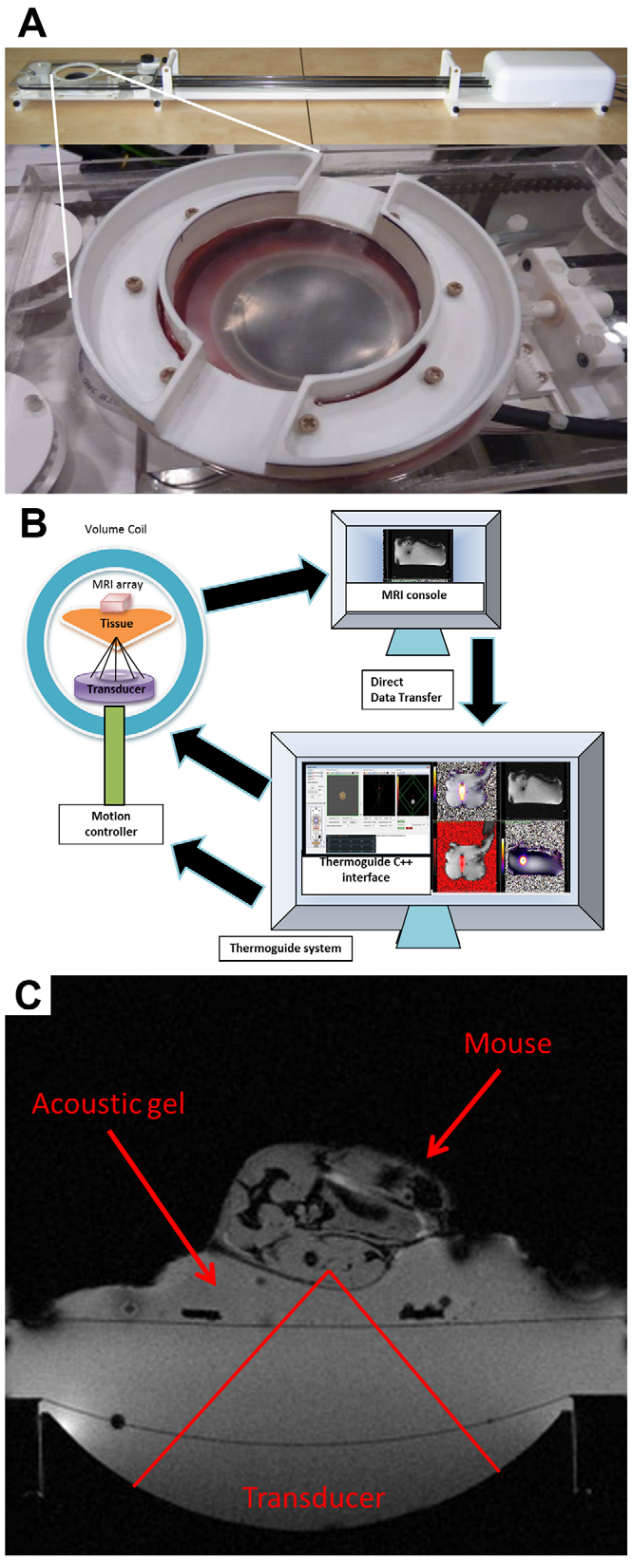
Experimental setup of the MRgFUS system. (a) A photograph of the MR compatible ultrasound insert (top) and a close up of the end of the insert in which the 16 element annular array is water coupled to an upper plastic membrane (white arrow) on which the sample or animal would rest. (b) Schematic of combined MRgFUS system for real-time feedback control of arbitrary heating trajectories. (c) Axial MR image illustrating the experimental setup utilized for *in vivo* experiments; the rat brain phased array was positioned on the mouse, but is not visible in the image due to decoupling and choice of TE (though the image was acquired with the volume resonator for both RF transmit and receive to facilitate better visualization of the entire experimental setup).

A MR compatible FUS transducer (IMASONIC SAS, Voray sur l'Ognon, France) for precise heating (16 element annular transducer, 3 MHz central frequency, 300 kHz bandwidth, 19 W acoustic peak power, 85° aperture, 48 mm diameter, 35 mm radius of curvature, adjustable focus depth, 1 mm×1 mm×2 mm focal spot size) with an embedded transducer positioning system (Image Guided Therapy, Pessac, France) facilitating accurate transducer trajectory execution (MR compatible piezoelectric motors, 30 mm horizontal positioning range, 11 mm vertical positioning range, 10 micrometer nominal positioning resolution, active transducer cooling system, see also Video S1). The annular array consists of ring elements on a spherically-curved surface with varying arc lengths between inner and outer radii resulting in approximately equal area elements with areas ranging from 1.089 cm^2^ for the innermost elements up to 1.167 cm^2^ for the outer-most elements (Table S1). The overall radiating area of the array is 17.84 cm^2^. The widths of the elements (in arc length) range from 0.775 mm for the outermost element up to 11.8 mm for the innermost element. The overall form of the FUS insert (Fig. 1a) was designed for optimal fit within the 154 mm internal diameter MR quadrature coil utilized for this study.

The array is driven and electrically monitored by a programmable 16-channel RF pulse transmitter (Image Guided Therapy, Pessac, France). The transmitted RF electrical power (including voltage, current and phase) to each element is logged continuously during the operation of the pulser.

### MRgFUS Software for Real-Time Closed Loop Feedback Control

Both the MR and FUS controllers were placed on a dedicated local network which allowed real-time, frame-by-frame transfer of non-reconstructed MRT data to the FUS controller for reconstruction by the provided Thermoguide software (Image Guided Therapy, Pessac, France) (Fig. 1b). The proportional, integral, and derivative (PID) feedback controller is based on the bioheat transfer equation (BHTE) in the following form [27], [28]:
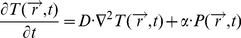
(1)where *T* is temperature, 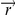 is spatial location, *t* is time, *∇* is the spatial gradient operator, *D* is the thermal diffusion in mm^2^/s, α describes the focal temperature increase in Kelvin per Joule of transmitted acoustic energy (units of K/J_AC_), and *P* is the applied acoustic power in W. The PID controller, based on Equation 1 and described elsewhere [27], was tested *in vitro* in a tofu phantom followed by *in vivo* validation using a Met-1 mouse tumor model with a predefined ultrasound beam trajectory and control points. Under PID control, sonication was performed for a total of 40 ms per control point, and the entirety of the heating trajectory was performed once per MR frame. Here a spiral trajectory was implemented for heating cylindrical volumes [29]. The thermal diffusion parameter, *D*, was set to a typical value of 0.1 mm^2^/s [30] for all experiments and we verified that the controller is insensitive to small variations of this parameter. The primary PID tuning parameter, α, was experimentally-determined by measuring the temperature increase for 1 W of acoustic power. Using this method, a value of α = 1.4 K/J_AC_ for tofu was determined for the system. To check this value of α, the PID controller was tuned by minimizing the standard deviation of the temperature during steady state control for single-point heating as a function of α (data not shown), and it was found that the experimentally determined α produced minimum deviations in the steady-state controlled temperature.

### FUS Beam Simulation

An acoustic model for the 16 element annular array was developed using Fast Object-oriented C++ Ultrasound Simulator (FOCUS, http://www.egr.msu.edu/focus-ultrasound/) run in MATLAB (v2011a, MathWorks, Inc., Natick, MA). FOCUS was chosen due to ease of implementation given the geometry of the annular array. The geometry of the array consists of a 35 mm radius of curvature with an outer diameter of 48 mm. The manufacturer specifications for the inner and outer radii of the annuli were used in the model to define the locations of each annulus on the spherical surface. The acoustic field was calculated according to the fast near-field method implemented for spherically-curved pistons in FOCUS. Field calculations assume linear propagation and infinite rigid baffled sources. Each annulus was defined as the superposition of a spherical piston with radius equal to the outer radius minus a spherical piston with radius equal to the inner radius. Although the array may be phased to focus at different depths, geometric focusing was assumed in this numerical study.

For thermal predictions, the acoustic field was simulated for a layered model of water and tofu. The acoustic and thermal properties of water and tofu [31], [32], [33], [34], [35], [36], [37], [38] used in the calculation are summarized in Table 1. The 100 µm thick high density polyethylene layer between the water and the tofu is thin enough to be neglected in the simulation. This is evident as there was no detectable loss within the polyethylene in hydrophone measurements. First, ultrasound propagation in water was simulated up to a depth of −5 mm inferior to the focal plane. The pressure field in this focal plane was calculated with a spatial resolution of λ/8 extending out to 2 array diameters. From this plane, the acoustic field was propagated through the tofu using the angular spectrum approach implemented in FOCUS with the spectral propagator method and angular restriction as detailed in [39]. The magnitude of the resulting pressure field was squared and normalized for thermal calculations. The transmission factor across the water-tofu interface was estimated to be 0.999 and thus negligible in terms of loss.

**Table 1:** Acoustic and thermal variables used during heating simulations for water and tofu.

	Water	Tofu	Reference
**Density (r)**	1000 kg m^−3^	1050 kg m^−3^	Water [31], tofu [36], [37], measured
**Specific Heat Capacity (C_p_)**	4180 J kg^−1^°C^−1^	4180 J kg^−1^°C^−1^	Water [32], tofu [36]
**Thermal Conductivity (K)**	0.60 W m^−1^°C^−1^	0.45 W m^−1^°C^−1^	Water [33], tofu [38]
**Acoustic Attenuation (a)**	0.225 Np m^−1^ (3.0 MHz)	23.6 Np m^−1^ (3.0 MHz)	Water [34], tofu [36], [37], measured
**Speed of Sound (c)**	1492.4 m s^−1^ (23.0°C)	1510.6 m s^−1^ (23.0°C)	Water [35],tofu [36], [37], measured

Thermal predictions were carried out using a finite-element model developed in COMSOL Multiphysics (v3.5, COMSOL, Inc., Burlington, MA). The two-layer experimental setup consisted of a water layer in thermal contact with a sample of tofu (silken style, extra firm, Mori-Nu, Torrance, CA). Due to the axial symmetry of the acoustic field, the thermal model was also axially-symmetric. The dimensions consist of a water layer 5 mm deep by 12.5 mm in radius and a tofu layer 25 mm deep by 12.5 mm in radius. The finite-element mesh spanning the 2-D cross-section contains 5184 triangular elements with an average area of 0.0603 mm^2^ and much smaller in the beam focal region (<<λ^2^). The boundary conditions are as follows: the left boundary at r = 0 mm is the axis of symmetry, the top, right and bottom boundaries are held at constant ambient temperature, and the interface at z = 5 mm assumes thermal continuity. Effects of acoustic streaming and resulting convection in the water path are neglected for the short-duration heating considered here. The acoustic intensity at arbitrary mesh node locations was determined by cubic spline interpolation from a table containing the normalized acoustic intensity field sampled at λ/8 in r, the radial dimension, and z, the depth dimension. The transient solver SPOOLES (SParse Object Oriented Linear Equations Solver) was used with strict time stepping not greater than 0.1 s, and the resulting simulated temperature distribution obtained after 15 s of heating for a peak-acoustic pressure of 1 MPa was calculated.

### FUS Beam Characterization

The FUS beam profile was measured in the lateral direction using a 0.5 mm active diameter Müller-Platte needle hydrophone (model HNS-0500, Onda Corporation, Sunnyvale, CA), which is specifically designed for measuring high acoustic field from highly focused sources [40]. The measurement was performed in a degassed water tank. The measured angular directivity of this hydrophone at 3 MHz corresponds to an effective aperture diameter of 0.578 mm, which may be used to accurately access acoustic pressures with the center 6 array elements turned on (corresponding to f/2.37). A linear fit (R^2^ = 0.999) to the pressures obtained from the first six elements resulted in a correction factor of 1.52±0.03 required to estimate the true pressure (assuming linear acoustics) at the geometric focus from the measured pressure for all 16 elements turned on. For highly focused arrays, the geometric focus corresponds closely to the true focus [41]. Standing wave issues were eliminated by making hydrophone measurements using 10 microsecond duration pulses (∼33 cycles) sufficiently away from reflecting boundaries.

**Figure 2:**
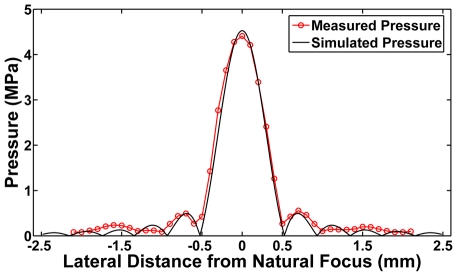
Comparison of measured and simulated pressure distributions of the annular array. Calculated pressure as function of distance derived from simulation of the 16 element annular array compared to the measured pressure in the lateral direction. The measured −6 dB beam width was 0.8 mm, which agreed well with simulated results.

For the 16-element annular array, the normalized radial beam profile in the focal plane obtained via simulations corresponded well with hydrophone measurements obtained in water (Fig. 2), each with a -6 dB beam width of 0.8 mm. For both simulations and measurements, the first side lobe occurred 0.7 mm from the focal spot center with intensity ∼−10 dB compared to the focal spot. The simulated −6 dB depth-of-focus of 2.3 mm was not verifiable by hydrophone due to the variable and limited directivity of the hydrophone over the angular range required for accurate measurement of this quantity for this f/0.73 array.

The acoustic power radiated by the array was measured using the absorptive radiation balance technique including the correction factor for focused sources [42]. For the electrical powers investigated in this paper, the efficiency of the array ranged from 37–38% for electrical powers ranging from 1.27 W up to 4.83 W.

### MRT

Temperature change measurement calculations were performed with the aid of Thermoguide, and subsequently validated offline in MATLAB (v2011a, The Mathworks, Natick, MA) using the raw MR data on a pixel by pixel basis according to the temperature dependence of the PRF [8]:
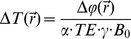
(2)where α = −0. 0101±0.0004 ppm/°C [2], [11] is the temperature-dependent electron screening constant, 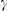 is the gyromagnetic ratio of ^1^H, 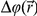 is the MR phase difference at the voxel 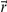, *TE* is the echo time, and 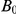 is the static magnetic field strength. The phase difference at each point was calculated via complex subtraction. The phase difference was obtained as follows [43]:
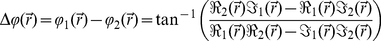
(3)where 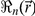 and 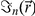 are the real and imaginary components of the *n^th^* complex image at the voxel 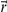, respectively. The calculated phase difference was then scaled to temperature using Equation 2 to yield the temperature difference, which was subsequently used to calculate the absolute temperature using the known temperature of the reference image. Correction for phase drift was made by fitting a polynomial to a small region away from the heated region yet still within the sample.

### MRT sequence comparison and validation

Echo Planar Imaging (EPI) facilitates rapid acquisition and has been implemented for measurement with the PRF method [44]. EPI at high field strengths can be particularly challenging due to magnetic susceptibility effects resulting in signal loss (reduced 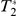). While single shot EPI (ssEPI) offers some additional temporal resolution compared to segmented EPI (segEPI), ssEPI is particularly challenging at 7T due to the short 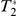, especially in the presence of the dielectric US transducer. Thus, we chose to examine segEPI sequences with and without GRAPPA, both of which still providing high temporal resolution. The SNR, temporal resolution, and temperature accuracy of GRE, and segEPI sequences (both with and without GRAPPA) were compared in a tofu phantom. Five sequences were compared (Table 2): A basic GRE without GRAPPA (GRE-0), a reduced TR GRE without GRAPPA (GRE-1), GRE with GRAPPA (GRE-2), segEPI without GRAPPA (seg-EPI-1), and segEPI with GRAPPA (seg-EPI-2).

**Table 2:** Comparison of GRE and segEPI sequences with and without GRAPPA.

	GRE-0	GRE-1	GRE-2	seg-EPI-1	seg-EPI-2
**TA per slice**	1000 ms	640 ms	370 ms	120 ms	100 ms
**TE/TR/FA**	4.5 ms/15.625 ms/30°	4.5 ms/10 ms/5°	4.5 ms/10 ms/5°	10 ms/30 ms/30°	10 ms/25 ms/30°
**GRAPPA**	1	1	2	1	2
**Partial Fourier**	1	1	4/5	1	4/5
**SNR_mag_**	137	77.2	48.9	20.7	15.4
**σ(T_Luxtron_−T_MR_)**	0.379°C	0.471°C	0.562°C	0.482°C	0.941°C

TA = acquisition time; TE = echo time; TR = repetition time; FA = flip angle; SNR = signal-to-noise ratio.

Sequence characterization and validation were performed in a tofu phantom. MR data were acquired using a 4 channel receive only phased array. Spatial resolution was fixed at 1 mm^3^ isotropic in all cases. Acquired MR data were reconstructed within the scanner software, and GRAPPA was utilized when PPI was implemented. Use of a phased array necessitated computation of the SNR as follows [45]:
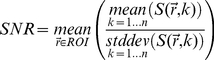
(4)where 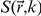 is the signal (intensity) of the voxel 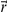 on the *k* ^th^ image in a series of *n* images. This method of SNR calculation was chosen to avoid assumptions regarding the statistical or spatial distribution of image noise [45].

For validation of MRT measurements, a fluoroptic temperature probe (Luxtron, Santa Clara, CA) was inserted and fixed in the tofu prior to sonication. The tip of the fluoroptic probe was localized via MRI using a fast gradient echo (GRE) sequence. A circle of radius 1.5 mm centered about the tip of the fluoroptic temperature probe was chosen as the heating trajectory to prevent the fluoroptic probe from being directly at the focal spot of the beam. Additionally, the fluoroptic probe diameter is 0.5 mm, thus smaller than a 1 mm^3^ voxel for adequate spatial sampling of the voxel corresponding to the MRT measurement irrespective of the temperature elevation distribution in plane of the trajectory.

Single point heating was not used in conjunction with the fluoroptic probe since the temperature measurements made with the fluoroptic probe in this combination would not yield an accurate temperature measurement because the FUS focal spot size is smaller than the tip of the fluoroptic probe. Moreover, if the focal spot is moved away from the tip of the fluoroptic probe, the temperature rise will be asymmetric about the fluoroptic probe if the FUS beam is not scanned. This will also result in fluoroptic temperature measurements that are not accurate. Thus, we chose to validate MRT against the fluoroptic probe in experiments where the FUS beam could be scanned about the probe to yield a more homogenous temperature within the region surrounding the probe. Moreover, it was determined that the mechanical movement of the transducer positioning system did not cause significant temperature measurement artifacts.

Temperature maps were derived using the acquired MR data and the time of acquisition was matched to the fluoroptic temperature probe data for comparison. The MRT measurements were compared to the corresponding fluoroptic temperature measurements to derive a mean temperature difference over time. Each compared MRT sequence was evaluated according to this procedure.

### Spatial Sampling of MRT Measurements

Previous work on spatial sampling in MRT [46] suggests the potential for errors in temperature change measurements if appropriate spatial resolution is not used. Indeed, the use of a 3 MHz, tightly focused ultrasound beam (the −3 dB intensity beam-width is 600 µm at the focus) ideally requires a spatial resolution of at least 300 µm during the transient heating phase. As the heating phase ends, thermal diffusion spreads the heated spot to permit accurate measurement with lower spatial resolution. The diameter of the fluoroptic temperature probe used in this study was greater than 300 µm, and therefore the study must account for spatial under-sampling of the temperature measurements during the transient heating phase. Moreover, the isotropic spatial resolution of the MR temperature maps (1 mm^3^) also produces spatial averaging. To model this effect, we included a simple 3-D spatial averaging filter in the simulated temperatures.

A tofu phantom was insonated at a single point using the experimental FUS system at 1.83 W acoustic power for 15 s to examine the effects of spatial resolution on the measured temperature. Single point insonation at the natural focus should yield the highest peak temperature over the smallest volume and would thus be best suited to examine potential spatial sampling inadequacies during transient heating. Voxel sizes of 0.5×0.5×0.5 mm^3^, 1×1×1 mm^3^, 2×2×2 mm^3^, and 1×1×2 mm^3^ (1 mm^2^ in-plane, slice thickness = 2 mm) were chosen as typical resolutions and voxel sizes used for rapid MRT imaging. MR data were acquired for a total of 5 minutes (including 60 s prior to sonication to evaluate 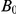 drift and ensure steady state of the gradient system).

### *In vitro* controlled heating

To evaluate the behavior of the PID controller, we first insonated a tofu phantom using sequence GRE-0 for imaging and real-time temperature calculations. An increase of 4°C above the initial ambient temperature was chosen as compatible with mild hyperthermia and was thus used as the target temperature for PID control. A spiral trajectory was chosen to provide more uniform heat distribution and a slow ramp up to target temperature was used to simulate the slow increase desired for *in vivo* applications.

### *In vivo* controlled heating

A total of 4 animals (female FVB mice, 6–10 weeks, 15–25 g, Charles River Laboratories, Wilmington, MA) were examined over the course of the study providing the ability to detect an ∼30% change with a power of 0.8 and alpha of 0.05. Met-1 tumors were transplanted bilaterally into the mammary fat pad. Mice were anesthetized with 3.5% isoflurane, maintained at 2.0–2.5% isoflurane, and placed on a customized holder in lateral recumbency allowing acoustic gel coupling of the transducer to the tumor via a central opening of 25 mm diameter in the center of mouse holder. A heated water circuit was integrated within the holder allowing the animal to be kept warm during the procedure. Rectal temperature of the mice was monitored via an MR compatible small animal monitoring and gating system (Model 1025, SA Instruments Inc., Stony Brook, NY). The US insert was placed into the bore of MR scanner (Fig. 1c) and the tumor was localized with a GRE sequence. Real-time temperature feedback was performed for a target temperature increase of 4°C above physiological temperature (as measured with a rectal temperature probe) using a predefined trajectory consisting of 20 control points such that the overall heated region was contained entirely within the viable tumor.

## Results

### Comparison of Simulated to Measured Heating

**Figure 3:**
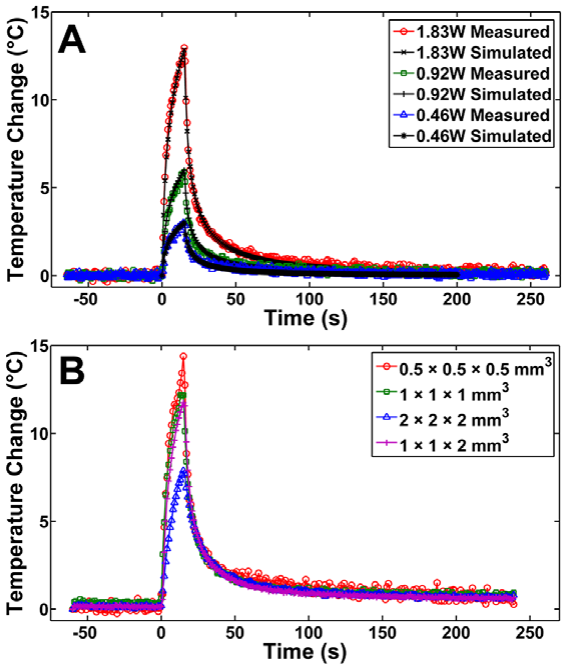
Temperature profiles for 15 s insonation compared to simulated results and evaluated for the effects of spatial sampling. (a) Temperature profile of a tofu phantom insonated for 15 s at 0.46 W, 0.92 W, and 1.83 W acoustic as measured by MR, compared to simulated temperature profiles for the same powers using a finite-element model. (b) Temperature profile of a tofu phantom insonated for 15 s at 1.83 W acoustic for MR spatial resolutions of: 0.5 mm^3^ isotropic, 1 mm^3^ isotropic, 2 mm^3^ isotropic, and 1×1 mm^2^ in-plane with 2 mm slice thickness. This demonstrates consistent temperature measurements at in-plane resolutions of at least 1×1 mm^2^.

The temperature profile produced in a tofu phantom insonated for 15 s at 0.46 W, 0.92 W, and 1.83 W was compared to the simulated temperature profiles for the same powers (Fig. 3a) demonstrating similar temporal profiles and linear scaling with power, except for the 1.83 W case, which required 7.5% more power over the linear scaling factor in order to achieve a good fit to the measurements. The MRT measured peak temperatures in the focal region at 15 s of heating in tofu were 3.0±0.3°C, 5.9±0.6°C, and 14.0±0.4°C for the 0.46 W, 0.92 W, and 1.83 W cases, respectively. The mean and standard deviation of the difference between the measured and predicted temperatures estimated over 200 s of the heating profiles shown in Fig. 3a were 0.05±0.1°C, 0.02±0.2°C, and −0.9±1.6°C for the 0.46 W, 0.92 W, and 1.83 W cases, respectively.

### Spatial Sampling of MRT Measurements

MRT measurements acquired with different spatial sampling were not significantly different for an MR spatial resolution as large as 1 mm^2^ in-plane, but MRT data acquired with a spatial resolution of 2 mm^3^ or greater yielded reduced peak temperatures (Fig. 3b). However, during the cooling phase, the measured temperatures were in good agreement across all resolutions examined. Peak temperatures were 14.4°C±0.6°C, 12.2°C±0.8°C, 11.7°C±1.1°C, and 7.9°C±0.7°C for 0.5 mm^3^, 1 mm^3^, 1×1×2 mm^3^, and 2×2×2 mm^3^ respectively. Computer modeling of the MRT spatial averaging of the predicted 3-D temperature distributions reduced the predicted peak temperatures at 15 s from 4.8°C to 3.0°C, 9.7°C to 6.0°C, and 20.9°C to 12.8°C for the 0.46 W, 0.92 W, and 1.83 W cases, respectively.

### MRT Sequence Comparison and Validation

Of the compared MRT sequences, magnitude SNR was 137±9.7 for the basic GRE sequence (GRE-0), 77.2±6.5 for a shortened GRE sequence (GRE-1) acquired in 640 ms while a segEPI (4 segments) sequence yielded a magnitude SNR of 20.7±2.2 in 120 ms without PPI. With a GRAPPA factor of 2 and partial Fourier = 4/5, the scan time was decreased to 100 ms in the segEPI sequence and the magnitude SNR was decreased to 15.4±1.9. Applying the same parameters to GRE-1 (Partial Fourier = 4/5 and GRAPPA = 2) decreased scan time to 370 ms and magnitude SNR to 48.9±4.2. Calculated temperature standard deviation followed the trend GRE-1<segEPI-1<GRE-2<segEPI-2 illustrating lower thermal precision for GRAPPA acquisitions.

**Figure 4:**
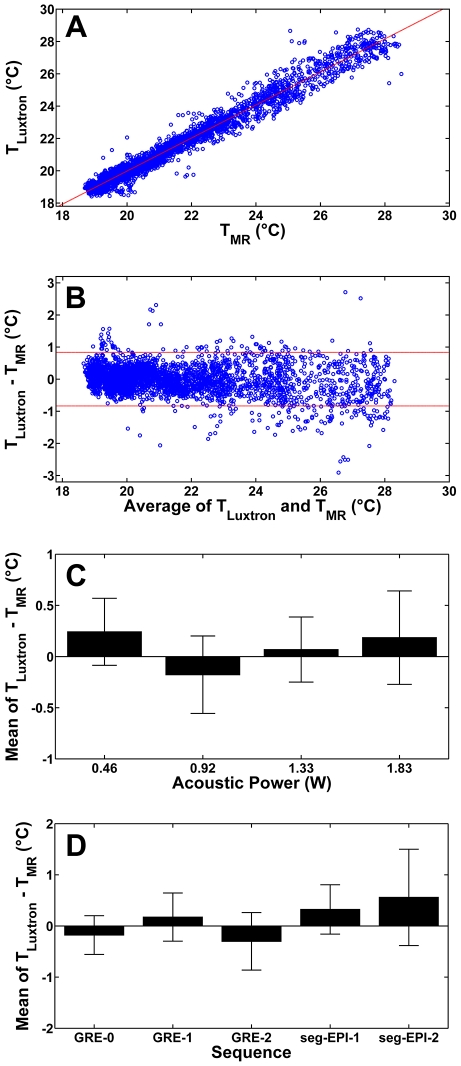
*In vitro* validation of MR-derived temperature measurements against a fluoroptic temperature probe. (a) Linear regression (dashed line) of MR-derived temperature (T_MR_) versus fluoroptic probe measured temperature (T_Luxtron_). The regression line is given by: T_Luxtron_ = 1.023·T_MR_−0.473°C (R^2^ = 0.969, p<0.05). (b) Bland–Altman plot comparing MR-derived temperatures (T_MR_) with corresponding fluoroptic probe-based temperatures (T_Luxtron_) with 95% confidence interval (1.96×SD of the differences) shown by the dashed lines. (c) The temporal mean of the difference between the MR derived temperature and the fluoroptic probe measured temperature (T_Luxtron_−T_MR_) for acoustic powers of 0.46 W, 0.92 W, 1.33 W, and 1.83 W averaged over 3 separate repetitions. (d) The difference between the spatiotemporal mean MR derived temperature and fluoroptic probe measured temperature for each MRT sequence examined. The precision of MR derived temperature measurements was within 1°C across all sequences.

MR temperature measurements (T_MR_) during a 3 mm diameter circular heating trajectory were compared to the corresponding fluoroptic probe measurements (T_Luxtron_) over the course of the experiment (Fig. 4a) and a linear fit was obtained with the relationship: T_Luxtron_ = 1.023·T_MR_−0.473°C (R^2^ = 0.969, p<0.05). Bland–Altman analysis [47] was also used (Fig. 4b) to provide an additional method of comparing T_MR_ with T_Luxtron_. The mean difference (averaged over all FUS power settings examined) was found to be 0.08°C, with a 95% limit of agreement (1.96×SD) of 0.83°C. Examined individually, by FUS power, the spatiotemporal mean of the difference of the temperature measured by the fluoroptic temperature probe and the temperature measured by the MR (using an ROI centered on the fluoroptic temperature probe place at the center of the circular trajectory) was within 0.2°C for each power setting (Fig. 4c) with a standard deviation less than 0.5°C in all cases. The mean difference between MR derived temperatures and fluoroptic measured temperatures was also compared across all sequences examined (Fig. 4d), and the thermal precision was found to be within 1°C in all cases.

### *In vitro* controlled heating

**Figure 5:**
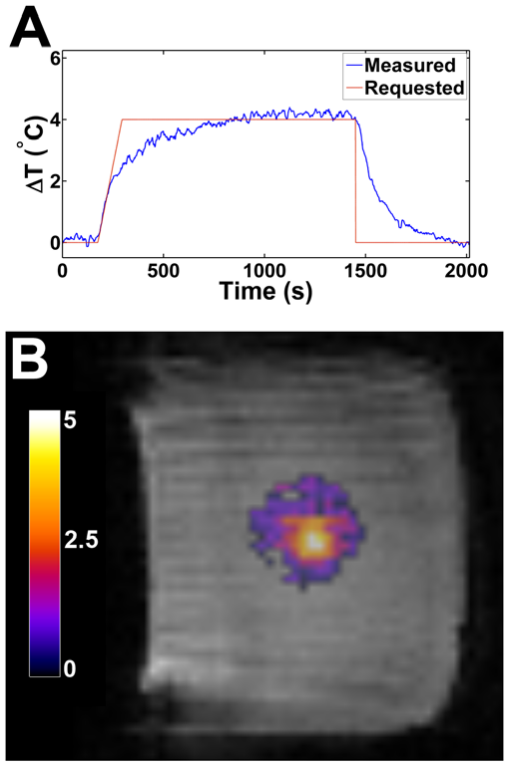
*In vitro* controlled heating results in a tofu phantom. (a) The temperature increase as measured by the MR (blue) versus the requested temperature increase (red) for a tofu phantom heated under PID control in a spiral pattern. (b) A single MR magnitude frame 15 minutes into the heating experiment illustrating the temperature increase (color overlay) on the magnitude image.

MR derived temperature change is compared to the requested temperature change during PID controlled heating (Fig. 5a) of a tofu phantom. At the plateau of the heating, the difference between the spatiotemporal mean temperature change and the requested temperature change was determined to be −0.14±0.32°C. This reflects the fact that the measured temperature change lags slightly behind the requested temperature change. A magnitude MR image frame with temperature overlay (Fig. 5b) acquired midway through the heating experiment provides an example of the operator view. Overall, the requested temperature was in good agreement with the measured temperature.

### *In vivo* controlled hyperthermia

**Figure 6:**
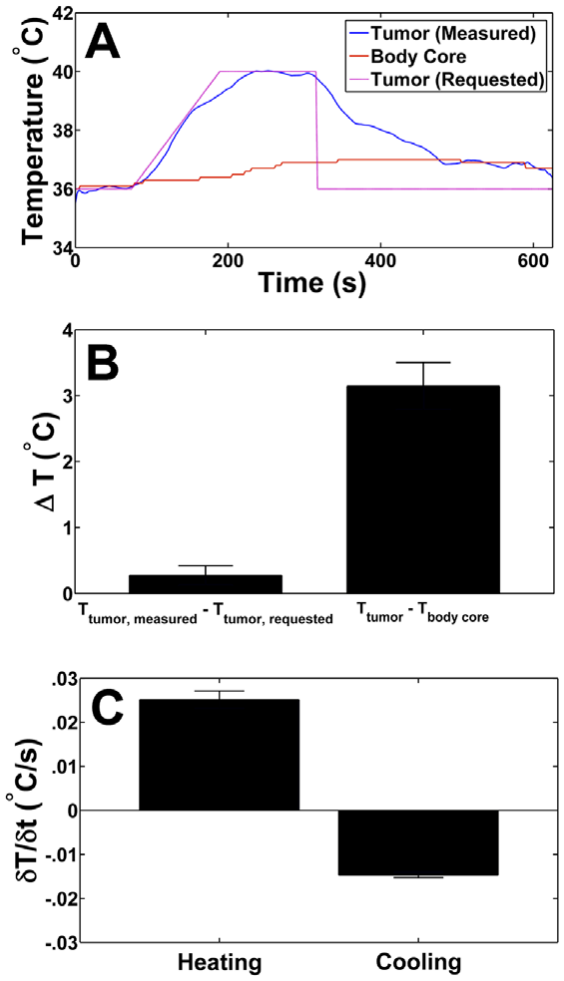
Temperature measurements during closed-loop heating *in vivo* in a Met-1 mouse tumor. (a) The MR derived temperature (blue) of an ROI consisting of the tumor region (identified via MR), compared to core body temperature (red) and the requested temperature (purple) over the course of the experiment for a target temperature increase of 4°C above starting temperature for a representative mouse. (b) The mean over all mice of the difference between the requested temperature and the measured temperature in the tumor at the heating plateau, and the mean of the difference between the tumor temperature and the core body temperature at the heating plateau. (c) The rate of change of tumor temperature during both the heating and cooling phases averaged across all mice. (d) MR magnitude image of an FVB mouse with Met-1 tumor illustrating the animal model used in this study.

**Figure 7:**
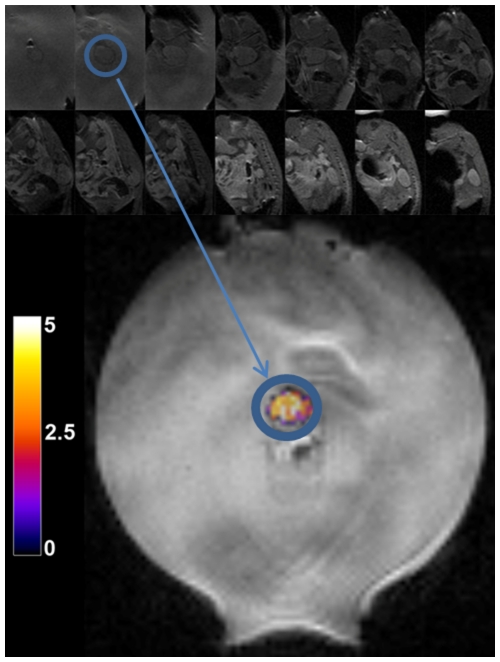
Localization and heating of Met-1 tumor. Spin echo multi-slice MR magnitude images (acquired in the coronal plane) used to localize the Met-1 tumor prior to heating (top two rows). The animal is positioned on an acoustic gel pad. The gel pad has a central hole in which the tumor sits. Following localization of the tumor from the image series, the appropriate coronal plane is used for imaging and real-time temperature control. Temperature is overlaid in color on a magnitude MR image mid-way through the heating sequence (bottom).

A representative case of measured temperature versus requested temperature along with the animal's core body temperature during the heating course is shown in Fig. 6a. The measured temperature at the plateau during local PID controlled heating of a Met-1 tumor to a target temperature of 4°C above physiological temperature yielded a measured spatiotemporal mean temperature increase of 3.1±0.4°C above the animals' core body temperature across all mice examined (Fig. 6b). The difference between the requested and measured temperature in the tumor at the time of the plateau was 0.27±0.15°C. The requested temperature increase was relative to the starting core body temperature, therefore slight global heating of the animal due to heat diffusion results in a slight decrease in the temperature difference at the plateau time. The rate of temperature change during the heating phase was approximately twice that of the cooling phase (Fig. 6c). A magnitude MR image with temperature overlaid in color is shown taken midway through a heating sequence. (Fig. 7, see also Videos S2 and S3)

## Discussion

High field MRT offers higher temperature sensitivity making it especially attractive for thermal therapies such as mild hyperthermia where precise temperature control is required. Our validation results suggest that for comparable spatial resolution and acquisition time, MRT at 7T promises a higher degree of thermal precision [48]. Conversely, for a similar thermal precision, high field systems can provide increases in temporal and spatial resolution. Other researchers have examined the suitability of high field scanners for temperature measurements using the temperature sensitivity of the PRF *in vivo* [49] in rodents during ablation with good results. Susceptibility effects at high field are significant with free breathing animals, and validation of the use of high field MRT was warranted to determine its suitability for the real-time monitoring of moderate temperature increases *in vivo*.

EPI is especially challenging at 7T, where shortened *T*_2_^*^ times result in signal loss, and image distortions may become severe. PPI reduces the impact of signal dropout in segEPI by decreasing the echo train length (ETL). While the ETL may be reduced in EPI without the use of PPI, doing so increases the acquisition time. Implementing PPI permits reduction of the ETL without compromising acquisition time. While we observed a decreased SNR and a decrease in the precision of thermal measurements, with this combination, we achieved temperature precision on the order of 1°C. The results of this study suggest that high field MRT offers good temperature precision and temporal resolution for real-time MRT during mild hyperthermia. Both fast GRE and segEPI sequences responded well to PPI using vendor provided GRAPPA algorithms with a reduction factor of 2.

A basic RF-spoiled GRE sequence yielded high SNR, good thermal precision, and reasonable temporal accuracy consistent with the overall requirements of mild hyperthermia. Moderate increases in temporal resolution are relatively easily achieved while maintaining reasonable SNR and thermal precision. EPI sequences offer rapid data acquisition, but the challenges of EPI at 7T are significantly increased from lower field implementations and as such we sought to determine the temperature precision afforded by this rapid imaging technique on a 7T scanner. With EPI sequences, a decrease in the precision of measured temperature was observed with an increase in image artifacts; however, the precision of the measured temperature for segEPI sequences was still arguably acceptable for most thermal therapy applications (see Table 2).

Errors due to inadequate spatial sampling in MRT have been investigated previously [46]; however, given the higher frequency FUS used in this study, it is prudent to investigate the impact of MR spatial resolution on measured temperatures. We found spatial resolutions of 1 mm^2^ or less at the FUS focal plane, and 2 mm or less in the propagation direction, did not yield significantly different peak or spatiotemporal mean temperatures. The lower spatial resolution requirement in the US propagation direction is consistent with simulations of the focal spot dimensions. A spatial resolution of 2 mm^3^ or greater yielded a lower peak temperature than that measured by either a fluoroptic probe or higher resolution MRT suggesting an inadequacy of spatial frequency sampling (partial volume effect). At lower spatial resolutions, the observed temperature is an average of the insonated region and the surrounding area where heat deposition is minimal during transient heating. Nevertheless, during less rapid temperature changes, such as those obtained from rapidly scanning a trajectory larger than the beam width or during cooling, a lower spatial resolution may be adequate.

PPI using the GRAPPA technique is feasible for increasing the temporal resolution of MRT when low reduction factors are used. While there are other PPI techniques offering higher image quality [12], [13], [14], the ease of implementation of the GRAPPA technique in combination with the low reduction factor facilitated the real-time implementation of PPI for MRT. Here, a reduction factor of 2 was used with temperature precisions on the order of 1°C.

Real-time temperature maps agreed with fluoroptic temperature measurements within 1.1°C in all cases examined (excepting peak temperature measurements made with low spatial resolutions specifically to investigate spatial sampling inadequacies).

MRT with real-time feedback has been validated by fiber optic temperature measurement in a tofu phantom and simulations of acoustic heating in tofu showed good agreement with MR temperature maps. An *in vitro* controlled heating experiment in tofu illustrated the ability of the system to maintain a moderate temperature increase for an extended period of time compatible with in vivo applications of mild hyperthermia. In an *in vivo* Met-1 tumor, the real-time PID feedback control is capable of maintaining the desired temperature with high accuracy, and although some global heating was observed, the predominant temperature increase was localized to the tumor region. In future work, this MR-HIFU system will be used to generate hyperthermia in tumors to enhance permeability and activate temperature-sensitive drugs in a controlled manner.

Overall, high field MRT has been validated with conventional fluoroptic temperature measurements, and PPI based on the GRAPPA technique has been validated for temperature accuracy with a reduction factor of 2. MR sequence comparisons demonstrated segEPI may be utilized on a 7T system with good temperature precision and high temporal resolution. The developed MRgFUS system has demonstrated feasibility for the delivery of a controlled thermal dose under real time PID control both in a tofu phantom and *in vivo* in a Met-1 mouse tumor. PPI acquisition with GRAPPA based reconstruction demonstrated adequate thermal accuracy, high temporal resolution, and was successfully implemented within the experimental MRgFUS system.

## Supporting Information

Video S1 Illustrative video of transducer positioning system during heating of *ex vivo* porcine muscle sonicated at 9.5 W acoustic power. Temperature (color overlay) is shown with the magnitude MR image.
(MP4)

Video S2 A Met-1 mouse tumor heated to 41°C with sonication under PID control to maintain the target temperature. The magnitude reconstructed image with real-time temperature overlaid is presented during sonication.
(MP4)

Video S3 A Met-1 mouse tumor heated to 39°C with sonication under PID control to maintain the target temperature. The magnitude reconstructed image with real-time temperature overlaid is presented during sonication. Data acquired with a 20 mm diameter circular surface coil for RF receive.
(MP4)

Table S1 Inner and outer radii, width and area of each element in the annular array used for this study.
(DOC)
