# Supplementary material for: Magnetic Resonance Thermometry at 7T for Real-Time Monitoring and Correction of Ultrasound Induced Mild Hyperthermia
Source: PLoS One. 2012 Apr 20;7(4):e35509. doi: 10.1371/journal.pone.0035509 (PMC3335017; doi:10.1371/journal.pone.0035509)
Supplement: Table S1 — Inner and outer radii, width and area of each element in the annular array used for this study. (DOC) [file pone.0035509.s004.doc]

| Element | Inner Radius (mm) | Outer Radius (mm) | Element width (mm) | Area (cm^2^) |
| --- | --- | --- | --- | --- |
| 1 | 0.00 | 5.87 | 11.7957 | 1.09022 |
| 2 | 6.07 | 8.41 | 2.3923 | 1.08867 |
| 3 | 8.61 | 10.37 | 1.8288 | 1.09069 |
| 4 | 10.56 | 12.02 | 1.5426 | 1.09447 |
| 5 | 12.21 | 13.47 | 1.3546 | 1.09294 |
| 6 | 13.65 | 14.77 | 1.2257 | 1.09442 |
| 7 | 14.95 | 15.97 | 1.1370 | 1.10457 |
| 8 | 16.15 | 17.08 | 1.0567 | 1.10325 |
| 9 | 17.26 | 18.12 | 0.9967 | 1.10795 |
| 10 | 18.29 | 19.10 | 0.9582 | 1.12561 |
| 11 | 19.27 | 20.02 | 0.9063 | 1.11870 |
| 12 | 20.18 | 20.89 | 0.8768 | 1.13138 |
| 13 | 21.06 | 21.73 | 0.8466 | 1.13819 |
| 14 | 21.89 | 22.52 | 0.8151 | 1.13724 |
| 15 | 22.67 | 23.28 | 0.8087 | 1.16740 |
| 16 | 23.43 | 24.00 | 0.7751 | 1.15497 |
